# Comparative evaluation of large language models for generating CAD-RADS 2.0-compliant diagnostic conclusions in cardiac CT reports

**DOI:** 10.1186/s13244-026-02285-6

**Published:** 2026-04-22

**Authors:** Giovanni Lorusso, Giorgio Ruscino, Alessia Spitaleri, Chiara Morelli, Sara Greco, Ilaria Villanova, Nicola Maria Lucarelli, Michele Mariano, Amato Antonio Stabile Ianora, Nicola Maggialetti

**Affiliations:** Interdisciplinary Department of Medicine, Section of Radiology and Radiation Oncology, University of Bari “Aldo Moro”, Bari, Italy

**Keywords:** Large language models (LLMs), Coronary computed tomography angiography (CCTA), CAD-RADS 2.0, Structured reporting, Artificial intelligence (AI) in medical reporting

## Abstract

**Objectives:**

Coronary computed tomography angiography (CCTA) has become a cornerstone in non-invasive CAD diagnosis and risk stratification. To standardize reporting and improve clinical decision-making, the CAD-RADS 2.0 system was introduced. This study evaluates the performance of four LLMs, GPT-4o, Gemini 2.0 Flash, DeepSeek V, and Copilot in generating CAD-RADS 2.0-compliant conclusions from standardized CCTA reports.

**Materials and methods:**

A total of 196 anonymized CCTA reports were retrospectively analyzed. Each LLM was prompted to provide CAD-RADS 2.0 classifications and follow-up recommendations. Ground truth labels were assigned by a senior radiologist. Performance metrics (accuracy, precision, recall, F1-score), execution times, and agreement (Cohen’s kappa) with expert interpretation were computed. Interobserver agreement between junior and senior radiologists was also assessed.

**Results:**

LLMs demonstrated good-to-excellent agreement with expert classifications: DeepSeek V (κ = 0.771), Copilot (κ = 0.761), GPT-4o (κ = 0.759), and Gemini 2.0 Flash (κ = 0.634). DeepSeek V achieved the highest accuracy (91.83%). Intra-model consistency was perfect (κ = 1). However, LLMs failed to assign CAD-RADS modifiers. ChatGPT-4o provided the most accurate follow-up recommendations (71.94%). All LLMs outperformed radiologists in execution time (3–9 s vs. 15–20 s; *p* < 0.05).

**Conclusions:**

Generic LLMs demonstrate promising performance in automating CAD-RADS 2.0 classification from CCTA reports. However, limitations in modifier assignment and recommendation accuracy highlight areas for refinement before clinical integration.

**Critical relevance statement:**

This study explores the potential of large language models to facilitate standardized CAD-RADS 2.0 reporting from coronary CT angiography, highlighting a possible avenue to support workflow efficiency and clinical decision-making in non-invasive coronary artery disease evaluation.

**Key Points:**

- LLMs demonstrated strong potential in automating CAD-RADS 2.0-compliant structured reporting for CCTA.
- LLMs could significantly enhance efficiency in radiological reporting.
- LLMs need further optimization before clinical integration.

**Graphical Abstract:**

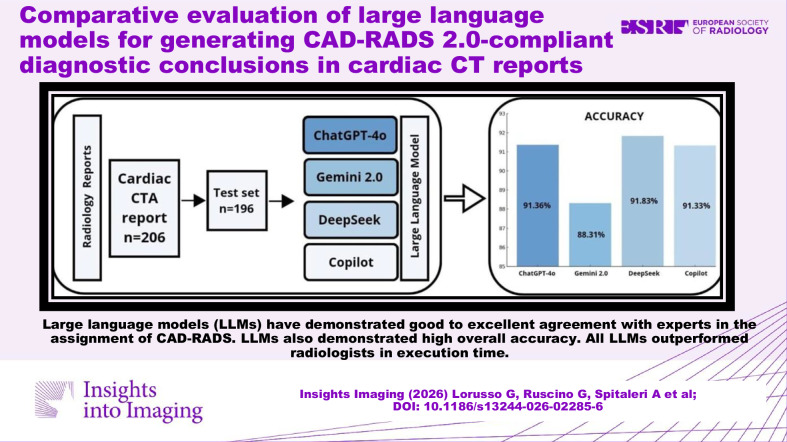

## Introduction

Coronary artery disease (CAD) is a leading cause of morbidity and mortality worldwide, underscoring the need for early detection and accurate risk stratification to improve patient outcomes. In 2022, CAD caused approximately 9.2 million deaths worldwide, with an age-standardized mortality rate of 108.8 per 100,000 people [1]. Coronary computed tomography angiography (CCTA) has established itself as a powerful, non-invasive imaging modality for diagnosing CAD [2]. CCTA is an effective, safe, and rapid technique that not only assesses stenosis severity but also differentiates plaque morphology and composition. It has demonstrated high sensitivity and a strong negative predictive value in patients with suspected stable CAD at intermediate-to-low risk, making it a reliable tool for detecting significant stenosis. Recent evidence suggests that CCTA also holds prognostic value [2, 3]. To ensure consistency in reporting and improve communication among healthcare providers, the Coronary Artery Disease Reporting and Data System (CAD-RADS) was introduced [4] by a multidisciplinary team of radiologists and cardiologists from leading professional societies. This structured reporting system standardizes the interpretation of CCTA findings, enhancing clarity in clinical decision-making and facilitating appropriate patient management. The updated version, CAD-RADS 2.0 [5], refines this framework by incorporating detailed classifications based on stenosis severity, plaque characteristics, and high-risk features, thereby improving risk stratification and guiding therapeutic strategies. Additionally, the inclusion of modifiers allows for a more comprehensive assessment by accounting for factors such as the presence of high-risk plaque (HRP), ischemia on functional testing (I), and the use of coronary bypass grafts (G), further refining patient management decisions (Table 1) [6].

**Table 1:** CAD-RADS 2.0: classification, indications for follow-up and modifiers [5]

CAD-RADS	Degrees of stenosis (%)	Interpretation	Follow-up indication
CAD-RADS **0**	0%	No evidence of coronary artery disease	No follow-up needed
CAD-RADS **1**	1–24%	Minimal non-obstructive stenosis	No follow-up needed
CAD-RADS **2**	25–49%	Mild stenosis	No follow-up needed
CAD-RADS **3**	50–70%	Moderate stenosis	Follow-up with functional test
CAD-RADS **4**	**A**: 70–99% with stenosis in 1 or 2 coronary vessels **B**: Stenosis > 50% in the left main or > 70% in 3 coronary vessels	Severe stenosis	**A**: Consider ICA or functional assessment **B**: ICA
CAD-RADS **5**	100%	Total occlusion or severe stenosis	Urgent follow-up with evaluation for surgery or angioplasty
CAD-RADS **N**	Nondiagnostic study	Nondiagnostic study	Additional assessments needed
**CAD-RADS modifiers**	**Description**		**Additional information**
**N**	Indicates that a study is nondiagnostic		If not all segments (> 1.5 mm diameter) are diagnostic modifier N should be listed
**HRP**	High-risk plaque		High-risk plaque features include: -low-attenuation plaque -positive remodeling -spotty calcification -napkin-ring sign If two or more of these features are present modifier “HRP” should be added to the CAD-RADS category
**I**	Ischemia		I+: indicates that CT-FFR demonstrates lesion-specific ischemia (≤ 0.75) or CTP reversible perfusion defect I−: indicates that CT-FFR is negative for lesion-specific ischemia (> 0.80) or CTP shows no reversible ischemia I±: indicates that CT-FFR (0.76–0.80) or CTP is borderline
**S**	Presence of stents		Total coronary plaque burden should also be added and is placed before the modifier S
**G**	Coronary artery bypass grafts		Importantly, a bypassed stenosis is not considered for CAD-RADS stenosis classification The total coronary plaque burden (combined assessment of native coronary arteries and bypass grafts) should also be added and is placed before the modifier G
**E**	Exceptions		The presence of non-atherosclerotic abnormalities should be added as modifier “E” to CAD-RADS score: • coronary artery dissection • anomalous origin of the coronary arteries • coronary artery (pseudo) aneurysm • vasculitis • coronary artery fistula • extrinsic coronary artery compression • arteriovenous malformation • other causes

The exponential growing volume of CCTA studies, coupled with the demand for rapid and reliable reporting, has led to the exploration of artificial intelligence (AI) as a potential solution; AI has demonstrated significant promise in supporting radiologists by automating repetitive tasks, enhancing image analysis, and assisting in lesion detection, radiology training and structured reporting [7–10]. Among AI-driven technologies, large language models (LLMs), a subset of AI specializing in natural language processing (NLP) based on Transformer architecture, have shown potential in automating various aspects of medical reporting, including the generation of diagnostic conclusions [11, 12]. LLMs’ ability to process complex medical texts and generate structured reports makes them attractive candidates for integration into radiological workflows, especially where structured reporting frameworks like CAD-RADS 2.0 play a pivotal role in ensuring consistent and accurate communication of findings.

Despite the potential of LLMs to streamline radiological reporting, their clinical accuracy and reliability remain overall underexplored; while LLMs have demonstrated competence in text summarization and interpretation [13, 14], their performance in medical imaging applications requires rigorous evaluation [15–17].

This study aims to address this gap by systematically comparing the accuracy, clinical relevance, and error profiles of four leading LLMs (GPT-4o, Gemini 2.0 Flash, DeepSeek V, and Copilot) in generating diagnostic conclusions from CCTA reports using CAD-RADS 2.0 as the standard framework. Additionally, this study aims to assess how freely available, non-fine-tuned LLMs interpret the textual content of radiology reports, summarize diagnostic impressions, and generate suggestions for further investigation based solely on report text.

## Materials and methods

### Study population

A retrospective analysis was performed on CCTA reports from 206 patients referred to our tertiary university center between January 2024 and October 2024.

The selection of reports was based on the use of a structured reporting format.

**Fig. 1:**
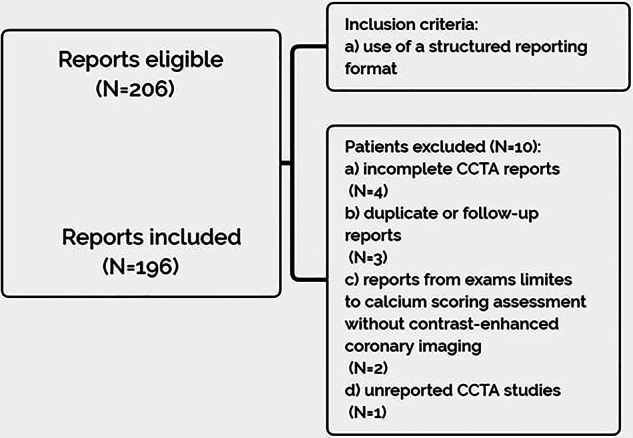
Flowchart of inclusion and exclusion criteria

Exclusion criteria included: (1) incomplete CCTA reports; (2) duplicate or follow-up reports; (3) reports from exams limited to calcium scoring assessment without contrast-enhanced coronary imaging; (4) unreported CCTA studies (Fig. 1).

The primary objectives were to evaluate the accuracy of four leading LLMs, GPT-4o, Gemini 2.0 Flash, DeepSeek V, and Copilot, in generating diagnostic conclusions based on CAD-RADS 2.0 and to assess how these models propose further investigations to support clinical decision-making.

### CAD-RADS assessment

The CAD-RADS coding framework is based on the assessment of stenosis severity, plaque burden, and modifiers.

Stenosis is expressed as a percentage considering the most severe data among the coronary vessels; CAD-RADS 0 indicates the absence of plaque or stenosis (0%) and, along with categories 1 and 2—representing minimal (1–24%) and mild (25–49%) stenosis, respectively—does not necessitate further diagnostic investigations. Conversely, additional functional assessments are considered in cases of moderate stenosis (50–70%), corresponding to CAD-RADS 3. The CAD-RADS 4 category is further subdivided into two groups: 4A, which includes single-vessel or two-vessel disease with severe stenosis (70–99%), and 4B, which includes left main stenosis > 50% or three-vessel disease with stenosis > 70%. In CAD-RADS 4A, the next step may involve invasive coronary angiography (ICA) or functional assessment, such as CT-FFR, CTP, or stress testing (exercise tolerance test, stress echocardiography, SPECT, PET, or cardiac MRI). In CAD-RADS 4B, ICA is recommended. Finally, ICA becomes mandatory in CAD-RADS 5, which describes a total vessel occlusion (100%). For category N, which indicates a nondiagnostic study, further evaluation is required.

The addition of descriptors from P1 to P4 is used to indicate increasing categories of plaque burden, assessed based on the Segment Involvement Score (SIS), the quantitative evaluation of total coronary plaque volume (measured by CCTA), or the Coronary Artery Calcium (CAC) score according to the Agatston method.

The modifiers include: “N,” indicating a nondiagnostic study due to motion artifacts, metallic interference, or other factors, which can be used either as a modifier or as a CAD-RADS category depending on the context; “HRP” i.e., high-risk plaque, defined by the presence of at least two abnormal features such as patchy calcifications, low attenuation, positive remodeling, and the napkin-ring sign; “I” indicates the result of an ischemia test (computed tomography fractional flow reserve, CT-FFR or stress computed tomography perfusion, CTP) if it was performed; “S” denotes the presence of at least one stent anywhere in the coronary system, while “G” indicates at least one coronary artery bypass graft; “E” represents exceptions, encompassing all non-atherosclerotic causes of coronary compression, such as dissections, anomalies of origin, aneurysms or pseudoaneurysms, vasculitis, fistulas, or extrinsic compression.

### Report analysis

Two junior and one senior cardiothoracic radiologist independently assigned CAD-RADS scores, modifiers, and recommendations for further investigations. The readers relied exclusively on the radiology report and were blinded to the imaging studies, the patient’s personal information, and clinical history. All reports were queried and retrieved using the institutional Radiology Information System (MedRIS Elefante system/Impax, AGFA Healthcare System).

The “I” modifier was not assigned, as this assessment was not available at our center. The time taken by each reader to generate a CAD-RADS score was also manually recorded using a stopwatch.

### LLMs report analysis

Analysis was carried out from 14 to 28 February 2025; we aimed to evaluate a heterogeneous sample of high-profile solutions representing the state of the art in next-generation general-purpose LLMs during that period. Selection was guided by both scientific and practical criteria, including potential accessibility and sustainability in clinical practice, widely adopted commercial reference models, an academically and industrially driven model with structured reasoning, and a model optimized for efficiency and speed.

Each report, excluding all potentially patient-identifying information, clinical history, and diagnostic conclusions, was input into the selected LLMs for analysis: ChatGPT (GPT-4o), Gemini 2.0 Flash, DeepSeek V-3, and Copilot, using the latest publicly available freeware versions at the time of the study.

Each LLM generated conclusions based on the same input prompt:

“Based on the following findings, provide a brief diagnostic conclusion in compliance with CAD-RADS 2.0 standards and suggest further investigations if necessary.”

For the analysis, we utilized our institutional standardized CCTA reports designed to systematically assess each coronary artery. The reports included details on examination technique, premedication, assessment of coronary artery origin and anomalies, calcium score measured using the Agatston method, plaque evaluation with details on presence, location, extent, eccentricity, characteristics, and degree of stenosis, presence of stents, grafts, bypass, HRP features, as well as valve assessment, aortic measurements, and additional findings. Coronary stenosis quantification was categorized as absent (0%), minimal (1–24%), mild (25–49%), moderate (50–70%), severe (70–99%), and occlusive (100%).

The models were not fine-tuned for this specific task, nor did they receive any feedback after assigning a CAD-RADS score or suggesting further examinations. A new session was initiated for each report, and all responses, including those where the LLMs failed to assign a score, were recorded.

In addition, the time taken by each LLM to generate a CAD-RADS score was also manually recorded using a stopwatch. This time was measured from the moment the report was entered into the LLM until the completed response was received.

### Statistical analysis

Statistical analysis was performed using SPSS software (version 30.0, SPSS Inc.).

Descriptive statistics such as frequencies, percentages, and means were calculated to summarize the distribution and characteristics of the study population and the model’s predictions.

Interobserver agreement between junior radiologists and expert radiologist was calculated with Cohen’s kappa coefficient (κ). For the evaluation of Cohen’s kappa, the benchmarks proposed by Landis and Koch were used, classifying the level of agreement as poor (0.00–0.20), fair (0.21–0.39), moderate (0.40–0.59), good (0.60–0.79), or excellent (> 0.80). To assess the consistency of each LLM, six randomly selected reports—one for each CAD-RADS category—were processed 100 times by each model, with Cohen’s kappa coefficient (κ) used to evaluate their agreement.

The ground truth CAD-RADS category was determined by the senior radiologist.

Agreement of ground truth and LLMs’ ratings was assessed by weighted Cohen’s kappa for two raters. Additionally, performance metrics such as accuracy, precision, recall, and F1-score were computed for each LLM. The F1 scores were determined by the harmonic mean of macro-averaged precision and macro-averaged recall of each CAD-RADS category.

The Friedman test was performed to assess variations in accuracy across models. A one-way ANOVA was performed to assess the presence of statistically significant differences in execution times among the four LLMs and between LLMs and radiologists. To further investigate which specific pairs of software differed significantly, a post hoc analysis was carried out using the Tukey HSD test. A *p*-value of less than 0.05 was considered statistically significant.

## Results

A total of 196 reports met the inclusion criteria out of the 206 reports retrospectively enrolled.

Of the 10 excluded reports, 4 were incomplete, 3 were duplicate or follow-up reports, 2 were performed only for calcium scoring, and 1 was unreported.

56 (28.57%) reports were classified by the senior radiologist, considered the ground truth, as CAD-RADS 0, 38 (19.39%) as CAD-RADS 1, 27 (13.78%) as CAD-RADS 2, 39 (19.90%) as CAD-RADS 3, 25 (12.76%) as CAD-RADS 4 (15 4A, 8 4B), and 4 (2.4%) as CAD-RADS 5 (Table 2).

**Table 2:** Distribution of CAD-RADS interpretations by radiologists and LLMs

	CAD-RADS 0	CAD-RADS 1	CAD-RADS 2	CAD-RADS 3	CAD-RADS 4	CAD-RADS 5	Modifiers
Senior radiologist	56	38	27	39	25 (15 A, 8 B)	4	70 HRP, 7 N, 12 S, 2 G, 5 E
Young radiologist	51	39	27	39	29 (20 A, 9 B)	2	70 HRP, 9 N, 12 S, 2 G, 11 E
GPT-4o	62	35	34	28	28 (10 A, 4 B)	7	2 N
Gemini 2.0	51	20	47	34	35 (6 A, 0 B, 7 C)	7	2 N
DeepSeek	58	24	34	34	38 (17 A, 13 B)	5	3 N
Copilot	51	40	26	30	40 (25 A, 7 B)	7	2 N

Interobserver agreement between the junior and senior radiologists for CAD-RADS classification showed an excellent level of concordance (90.31%; 177/196) with a Cohen Kappa coefficient (κ) of 0.874.

Modifier HRP was identified in 70 reports, modifier S in 12, modifier G in 2 by both young and senior radiologists (100% agreement, κ = 1).

5 modifiers “E” were assigned by senior radiologist and 11 by young radiologists, with a κ of 0.611 and 96.94% of agreement rate, revealing a good concordance.

7 modifiers “N” were assigned by senior radiologist and 9 by young radiologists, with an agreement rate of 97.45% and κ of 0.740, revealing a good concordance.

To assess intra-model consistency, each LLM processed six randomly selected reports, one for each CAD-RADS category, 100 times. All models consistently provided the same classification across all iterations (κ = 1).

Weighted Cohen’s kappa analysis for agreement with the ground truth CAD-RADS classification yielded the following values: GPT-4o (κ = 0.759), Gemini 2.0 Flash (κ = 0.634), DeepSeek V (κ = 0.771), and Copilot (κ = 0.761), indicating good to excellent agreement.

**Fig. 2:**
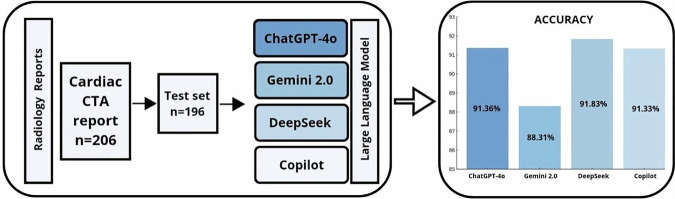
Study workflow and accuracy results of comparative analysis of four LLMs for generating CAD-RADS 2.0-compliant diagnostic conclusions in CCTA reports

The accuracy, precision, recall, and F1-score for each LLM in assigning CAD-RADS categories are summarized in Table 3. DeepSeek V and ChatGPT-4o demonstrated the highest overall accuracy (91.83% and 91.36%), followed by Copilot (91.33%) and Gemini 2.0 Flash (88.31%). The differences in accuracy were statistically significant, with a *p*-value = 00002 (Fig. 2).

**Table 3:** Performance metrics of LLMs in CAD-RADS classification

Model	Accuracy	Precision	Recall	F1-Score	Weighted kappa
GPT-4o	91.36%	56.57%	51.67%	57.74%	0.759
Gemini 2.0	88.31%	52.89%	36.94%	42.53%	0.634
DeepSeek	91.83%	60.23%	53.47%	54.63%	0.771
Copilot	91.33%	6.85%	55.38%	54.58%	0.761

Out of 196 reports, 12 included information regarding stents, 2 regarding coronary artery bypass graft surgery. None of the LLMs added any modifiers S or G.

LLMs exhibited variable accuracy in recommending further investigations based on CAD-RADS categories. In 71.94% of cases, ChatGPT-4o correctly identified the appropriate follow-up investigations, as determined by clinical guidelines; DeepSeek V followed with 68.37%, then Copilot (60.71%) and Gemini 2.0 Flash (47.96%). The accuracy of AI-generated recommendations was highest for CAD-RADS 0 cases (73.21–92.86%) and lowest for CAD-RADS 2 cases (18.85–48.15%), with frequent discrepancies in recommendations for invasive coronary angiography.

**Fig. 3:**
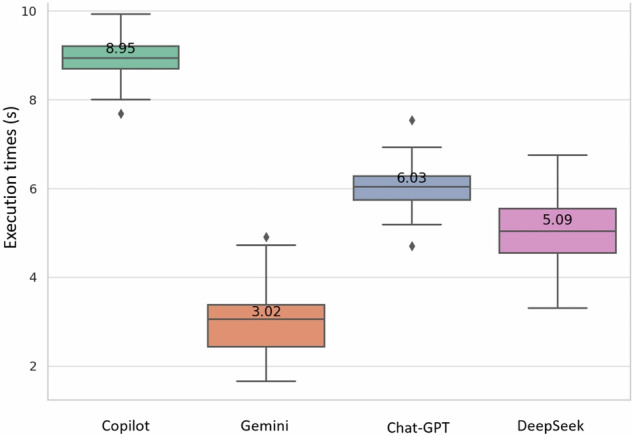
Box and whiskers plot of LLMs task completion times

Gemini 2.0 Flash was the fastest of all the models tested, with an average task completion time of 3 s, followed by DeepSeek V at 5 s, ChatGPT-4o at 6 s, and Copilot at 9 s. This difference was statistically significant (*p* < 0.05). All LLMs demonstrated faster performance compared to both junior and senior radiologists, who required 20 and 15 s, respectively (*p* < 0.05) (Fig. 3).

## Discussion

Coronary artery disease (CAD) remains a leading cause of morbidity and mortality worldwide [1]. Early detection and precise risk stratification are therefore essential to improve patient outcomes. In this context, CCTA has emerged as a powerful, non-invasive imaging tool for diagnosing CAD [2], while CAD-RADS enhances consistency and communication among healthcare providers [4]. As the volume of imaging studies continues to rise exponentially, radiologists face increasing pressure to maintain efficiency without compromising diagnostic accuracy. The intricate nature of CCTA analysis, combined with the need for fast and accurate reporting, has sparked growing interest in AI as a possible solution to assist in addressing these challenges. In particular, LLMs have shown promising potential in interpreting radiology reports, with generic models being publicly available and requiring no additional training costs or specialized expertise. Despite this advantage, selecting the most suitable model from the wide range of publicly available LLMs remains a challenge [17] and imposes inherent technical constraints, including throttling, stochastic output variability, and limited transparency regarding training data and updates. Using a standardized prompt and benchmarking against an expert radiologist on a monocentric dataset, we observed results that were internally consistent, indicating potential reproducibility that warrants further validation.

This study aims to conduct a comparative evaluation of four LLMs, ChatGPT, Gemini, DeepSeek, and Copilot, to assess their ability to generate CAD-RADS 2.0-compliant diagnostic conclusions in CCTA reports and appropriate recommendations for further investigations. The selection of models was guided by criteria such as accessibility, adoption, functional diversity, and relevance in the academic field, with the aim of conducting a meaningful comparative analysis of solutions that reflected the cutting edge of next-generation generalist freeware LLMs during the study period. According to the literature, each of these LLMs exhibits differences in terms of technical features and performance; in particular, ChatGPT-4o stands out for its high-quality language generation and versatility; DeepSeek V3 delivers excellent results in coding tasks; Gemini 2.0 Flash introduces unique multimodal capabilities, and Copilot represents a highly optimized solution for automatic code generation in production environments [14].

Interobserver agreement between junior and senior radiologists for CAD-RADS classification highlights a strong level of consistency especially for the most frequently assigned modifier, HRP (κ = 1); agreement rates for those with less clear definitions or more subjective interpretations such as E (κ = 0.611) and N (κ = 0.740) suggest good agreement, although slightly lower than HRP, indicating that some modifiers may be more prone to clinical experience. In our study, the modifier “I” was not assigned and studied, as this assessment is not available at our center.

The study demonstrated a good-to-excellent level of agreement between LLM-generated CAD-RADS classifications and the ground truth as determined by an experienced radiologist. However, the dataset included a predominance of CAD-RADS 0–2 cases, which are inherently less complex and may have contributed to higher overall performance metrics. Some differences were noted between the different LLMs: DeepSeek V (κ = 0.771) demonstrated the highest agreement, followed by Copilot (κ = 0.761) and GPT-4o (κ = 0.759), while Gemini 2.0 Flash (κ = 0.634) showed relatively lower agreement. In terms of performance, DeepSeek V achieved the highest accuracy (91.83%), followed by ChatGPT-4o (91.36%), Copilot (91.33%), and Gemini 2.0 Flash (88.31%). The “4C” category generated by one LLM is not defined within the official CAD-RADS framework and therefore represents an incorrect extrapolation rather than a valid classification. While each model exhibited perfect intra-model consistency (κ = 1), inter-model variability underscored differences in training data and architecture.

A detailed error analysis of LLMs thus highlighted inaccuracies in the assignment of high CAD-RADS categories, in the handling of modifiers, and discrepancies in clinical recommendations; in fact the LLMs exhibited reasonable accuracy in assigning primary CAD-RADS categories but they systematically failed to identify and include modifiers such as HRP, S, or G. This limitation is clinically significant, as modifiers are integral to full CAD-RADS 2.0 compliance and directly influence downstream management decisions; for example, HRP signals high-risk plaque that may warrant intensified medical therapy or closer follow-up, S indicates a stent guiding the assessment of restenosis, and G denotes a coronary graft influencing imaging evaluation and potential interventional planning. This highlights a key constraint on the real-world applicability of LLM-generated reports. A crucial component of structured reporting is not only classification but also appropriate recommendations for further investigations. Among the evaluated models, ChatGPT-4o achieved the highest accuracy (71.94%), followed by DeepSeek V (68.37%), while Gemini 2.0 Flash achieved the worst (47.96%). Notably, all models performed well in cases classified as CAD-RADS 0 (73.21–92.86% accuracy), likely due to the straightforward nature of recommendations in the absence of coronary stenosis. However, accuracy declined significantly for CAD-RADS 2 cases (18.85–48.15%), where discrepancies often arose regarding the need for further functional assessments. Similarly, CAD-RADS 4 cases presented challenges, with models occasionally underestimating the necessity of invasive coronary angiography. These findings underscore the importance of clinical oversight when incorporating LLMs into radiological workflows, particularly for intermediate-risk cases where management decisions are nuanced.

In this study, we also compared the task completion times of four LLMs, with Gemini 2.0 Flash emerging as the quickest model among them. In contrast, both junior and senior radiologists took longer to complete tasks than any of the LLMs. These findings highlight the significant potential of LLMs to transform radiological workflows, streamlining diagnostic processes and reducing turnaround times for radiological reports. By automating repetitive or time-consuming tasks, LLMs may help radiologists to focus more on complex cases and decision-making, although the models generate outputs faster than radiologists, speed alone does not compensate for reduced accuracy in cases where diagnostic guidance would be most impactful. Despite the promising results, this study has several limitations. First, the retrospective design and the small sample size in high CAD-RADS categories inherently restrict the generalizability of the findings, as the study population may not fully represent real-world clinical variability (selection and representativeness bias). Another key limitation is the reliance on structured reports, which ensures uniformity but does not account for the variability in free-text reporting commonly encountered in clinical practice. Furthermore, the study did not assess the impact of LLM-generated reports on patient outcomes. Finally, the models were evaluated in isolation without real-time feedback or iterative refinement or prompt engineering, which differs from how AI tools are typically integrated into clinical workflows. The absence of image data, clinical history, and model fine-tuning inherently limits the capacity of this study to evaluate true diagnostic reasoning or complex clinical decision-making. As such, the findings should be interpreted within the context of a text-only report interpretation task. While the models generated plausible summaries and recommendations, this does not imply readiness for integration into radiological workflows without substantially more sophisticated multimodal and clinically contextualized approaches. Future research should, in fact, take into account the rapidly evolving nature of LLMs, ensuring their safe and effective application in generating diagnostic conclusions.

The novelty of our study lies in the application of LLMs to a highly structured and realistic clinical context, using an innovative experimental setup. These results highlight the feasibility of using LLMs to support radiologists in daily workflows, improving standardization and reporting consistency, but the translational impact on clinical practice and patient outcomes remains to be established.

This study did not include a clinical validation involving cardiologists or an assessment of workflow-related outcomes such as reading time or changes in final CAD-RADS scoring. Future prospective studies incorporating blanking periods and time-based metrics are warranted to evaluate the clinical impact of LLM-generated CAD-RADS classifications. With further development, LLMs could be integrated into routine clinical practice, enhancing diagnostic safety and efficiency. Training on larger datasets and combining with specialized logic modules could improve modifier classification and follow-up accuracy. While current generalist LLMs primarily process text, multimodal research paves the way for integrating images and clinical data. Dynamic, updatable models accessing real-time data could ensure consistency with the latest guidelines and integration with PACS/RIS systems. Ultimately, LLMs may serve as intelligent radiologist assistants, providing interactive feedback, highlighting high-risk cases, and suggesting additional controls or follow-up.

In summary, while building on established tools, our work provides concrete evidence of the clinical and operational potential of LLMs, addressing a previously underexplored gap in the literature, and demonstrates preliminary validity with a methodologically solid basis for further evaluations and updates.

## Conclusion

LLMs such as GPT-4o, Gemini 2.0 Flash, DeepSeek V, and Copilot show preliminary potential in generating CAD-RADS 2.0-compliant structured reports from CCTA text data. DeepSeek V and GPT-4o demonstrated higher classification accuracy, and GPT-4o performed relatively better in suggesting follow-up investigations. However, limitations, including incomplete modifier assignment, lower accuracy in higher-risk cases, and inconsistencies in clinical recommendations, indicate that these models are not yet ready for direct clinical application. Future work involving model fine-tuning, integration of multimodal data, and hybrid AI-human workflows will be necessary to evaluate whether LLMs can meaningfully support radiological reporting and improve efficiency while maintaining patient safety.

## Data Availability

The datasets used and/or analyzed during the current study are available from the corresponding author on reasonable request.

## Abbreviations

AI: Artificial intelligence
CAD: Coronary artery disease
CAD-RADS: Coronary artery disease reporting and data system
CCTA: Coronary computed tomography angiography
CT: Computed tomography
CT-FFR: Computed tomography-derived fractional flow reserve
CTP: Computed tomography myocardial perfusion imaging
GPT: Generative pre-trained transformer
HRP: High-risk plaque
ICA: Invasive coronary angiography
LLM: Large language model

## References

[1] Stark B, Johnson C, Roth GA (2024) Global prevalence of coronary artery disease: an update from the Global Burden of Disease Study. J Am Coll Cardiol 83:2320

[2] Mallus MT, Prati F (2019) Utilizzo appropriato dell’angiografia coronarica con tomografia computerizzata nella cardiopatia ischemica: indicazioni e limiti. G Ital Cardiol 20:409–416. 10.1714/3190.31684 10.1714/3190.31684 31320762

[3] Abdelrahman KM, Chen MY, Dey AK et al (2020) Coronary computed tomography angiography from clinical uses to emerging technologies: *JACC* state-of-the-art review. J Am Coll Cardiol 76:1226–1243 32883417 10.1016/j.jacc.2020.06.076 PMC7480405

[4] Cury RC, Abbara S, Achenbach S et al (2016) CAD-RADS™: coronary artery disease–reporting and data system: an expert consensus document of the Society of Cardiovascular Computed Tomography (SCCT), the American College of Radiology (ACR) and the North American Society for Cardiovascular Imaging (NASCI). Endorsed by the American College of Cardiology. J Am Coll Radiol 13:1458–1466.e9. 10.1016/j.jacr.2016.04.024 27318576 10.1016/j.jacr.2016.04.024

[5] Cury RC, Leipsic J, Abbara S et al (2022) CAD-RADS™ 2.0—2022 coronary artery disease-reporting and data system: an expert consensus document of the Society of Cardiovascular Computed Tomography (SCCT), the American College of Cardiology (ACC), the American College of Radiology (ACR), and the North America Society of Cardiovascular Imaging (NASCI). JACC Cardiovasc Imaging 15:1974–2001. 10.1016/j.jcmg.2022.07.002 36115815 10.1016/j.jcmg.2022.07.002

[6] Xie JX, Cury RC, Leipsic J et al (2018) The coronary artery disease-reporting and data system (CAD-RADS): prognostic and clinical implications associated with standardized coronary computed tomography angiography reporting. JACC Cardiovasc Imaging 11:78–89. 10.1016/j.jcmg.2017.08.026 29301713 10.1016/j.jcmg.2017.08.026

[7] Parillo M, Vaccarino F, Beomonte Zobel B, Mallio CA (2024) ChatGPT and radiology report: potential applications and limitations. Radiol Med 129:1849–1863. 10.1007/s11547-024-01915-7 39508933 10.1007/s11547-024-01915-7

[8] Neri E, Aghakhanyan G, Zerunian M et al (2023) Explainable AI in radiology: a white paper of the Italian Society of Medical and Interventional Radiology. Radiol Med 128:755–764. 10.1007/s11547-023-01634-5 37155000 10.1007/s11547-023-01634-5 PMC10264482

[9] Saliba T, Ferrari J, Pozzessere C, Rotzinger D, Fahrni G (2025) Can advanced large language models support radiology training? A performance assessment of DeepSeek R1. Eur J Radiol Artif Intell 3:100024

[10] Arribas Anta J, Moreno-Vedia J, García López J et al (2025) Artificial intelligence for detection and characterization of focal hepatic lesions: a review. Abdom Radiol 50:1564–1583. 10.1007/s00261-024-04597-x 10.1007/s00261-024-04597-x 39369107

[11] Bhayana R (2024) Chatbots and large language models in radiology: a practical primer for clinical and research applications. Radiology 310:e232756. 10.1148/radiol.232756 38226883 10.1148/radiol.232756

[12] Kotkar AD, Mahadik RS, More PG, Thorat SA (2024) Comparative analysis of transformer-based large language models (LLMs) for text summarization. In: Proceedings of the 2024 1st international conference on advanced computing and emerging technologies (ACET), Ghaziabad, pp 1–7, 10.1109/ACET61898.2024.10730348

[13] Silbergleit M, Tóth A, Chamberlin JH et al (2024) ChatGPT vs Gemini: comparative accuracy and efficiency in CAD-RADS score assignment from radiology reports. J Imaging Inform Med. 10.1007/s10278-024-01328-y 10.1007/s10278-024-01328-y PMC12343400 39528887

[14] Liu Z, Zhong A, Li Y et al (2023) Tailoring large language models to radiology: a preliminary approach to LLM adaptation for a highly specialized domain. Mach Learn Med Imaging 14348:464–473

[15] Mallio CA, Sertorio AC, Bernetti C, Beomonte Zobel B (2023) Large language models for structured reporting in radiology: performance of GPT-4, ChatGPT-3.5, Perplexity and Bing. Radiol Med 128:808–812. 10.1007/s11547-023-01651-4 37248403 10.1007/s11547-023-01651-4

[16] Rahman A, Mahir SH, Tashrif MTA et al (2025) Comparative analysis based on DeepSeek, ChatGPT, and Google Gemini: Features, techniques, performance, future prospects. Syst Soft Comput 7:200396. 10.1016/j.sasc.2025.200396

[17] Mallio CA, Bernetti C, Sertorio AC, Beomonte Zobel B (2023) Large language models and structured reporting: never stop chasing critical thinking. Radiol Med 128:1445–1446. 10.1007/s11547-023-01711-9 37660320 10.1007/s11547-023-01711-9

